# Eugenol β-Amino/β-Alkoxy Alcohols with Selective Anticancer Activity

**DOI:** 10.3390/ijms23073759

**Published:** 2022-03-29

**Authors:** Cláudia Teixeira, Renato B. Pereira, Nuno F. S. Pinto, Catarina M. M. Coelho, Maria José G. Fernandes, António G. Fortes, Maria S. T. Gonçalves, David M. Pereira

**Affiliations:** 1 REQUIMTE/LAQV, Laboratory of Pharmacognosy, Department of Chemistry, Faculty of Pharmacy, University of Porto, R. Jorge Viterbo Ferreira 228, 4050-313 Porto, Portugal; pg40748@alunos.uminho.pt (C.T.); rjpereira@ff.up.pt (R.B.P.); 2 Centre of Chemistry, Department of Chemistry, University of Minho, Campus of Gualtar, 4710-057 Braga, Portugal; nuno_pinto1993@hotmail.com (N.F.S.P.); pg40075@alunos.uminho.pt (C.M.M.C.); fernandesmjgf@gmail.com (M.J.G.F.); gilf@quimica.uminho.pt (A.G.F.)

**Keywords:** eugenol, β-amino alcohols, β-alkoxy alcohols, cytotoxicity, apoptosis, anticancer

## Abstract

Eugenol, 4-allyl-2-methoxyphenol, is the main constituent of clove essential oil and has demonstrated relevant biological activity, namely anticancer activity. Aiming to increase this activity, we synthesized a series of eugenol β-amino alcohol and β-alkoxy alcohol derivatives, which were then tested against two human cancer cell lines, namely gastric adenocarcinoma cells (AGS) and lung adenocarcinoma cells (A549). An initial screening was performed to identify the most cytotoxic compounds. The results demonstrated that three β-amino alcohol derivatives had anticancer activity that justified subsequent studies, having been shown to trigger apoptosis. Importantly, the most potent molecules displayed no appreciable toxicity towards human noncancer cells. Structure-activity relationships show that changes in eugenol structure led to enhanced cytotoxic activity and can contribute to the future design of more potent and selective drugs.

## 1. Introduction

Cancer currently remains one of the leading causes of death, the global burden of cancer having increased in the last years as a consequence of higher life expectancy. In 2020 there were nearly 10 million deaths associated with cancer, with the highest mortality being associated with lung, colorectal, liver, stomach, and breast cancers [1]. Depending on the stage of development and the etiology involved, anticancer therapeutic strategies rely on chemotherapy, radiotherapy, and immunotherapy approaches. According to the latest studies, nearly 25% of chemotherapy drugs are directly obtained from plants, with an additional 25% of the molecules being naturally inspired [2].

The capacity of tumor cells to escape from programmed cell death pathways such as apoptosis is a hallmark of most types of cancer [3,4]. Various molecules are involved in apoptotic pathways, and caspases, a group of cysteine aspartic acid proteases, are crucial for triggering the intrinsic and extrinsic apoptosis pathways [5,6]. Although tumor cells lose proapoptotic components, surrogate ones can still be active. For this reason, new therapies that reactivate the apoptotic defense mechanism could be a great promise for cancer therapy [3,4].

Eugenol (4-ally-2-methoxyphenol) is a phenylpropanoid that can be found in several essential oils. *Syzygium aromaticum* (L.) Merr. & L.M. Perry is one of the most common sources of eugenol, which is usually obtained through steam distillation or hydrodistillation [7,8]. Eugenol has shown biological properties such as anti-inflammatory, antioxidant, antimicrobial, antitumorigenic, and cytotoxic activities [7,9,10]. It has been reported to possess antiproliferative effects in several cancer cells as well as being shown to activate mitochondrial apoptosis pathways in mast and leukemia cells and in rat models of gastric carcinogenesis and activates both apoptotic pathways in gastric cells (AGS) [11,12]. Moreover, eugenol has a good structure for use as a starting molecule due to its ready availability, so several derivatives have already been obtained and proven to enhance biological properties in relation to eugenol, namely anticancer activity [7,13]. Alterations such as alkylation of the free phenol of eugenol and attachment of a trioxygenated aromatic ring to the end of the three-carbon side chain result in synthetic compounds cytotoxic to breast cancer as well as in chemopreventive agents [7]. Additionally, nitro-derivative molecules presented higher cytotoxicity than eugenol against androgen-insensitive prostate cancer cells (DU-145) and oral squamous carcinoma cells (KB) [14].

In the present work, several eugenol derivatives—β-amino alcohols and β-alkoxy alcohols—were synthesized with the aim of improving the anticancer capacity of the parent molecule. With this aim in view, we evaluated the impact of these chemical structures on the activity profile of this family of molecules against cancer cells, having also benchmarked them against the original structures. We were also interested in assessing whether the chemical space of these molecules could be mapped and used in the future to guide future synthesis of increasingly potent molecules.

## 2. Results and Discussion

### 2.1. Eugenol β-Amino Alcohol Derivatives Are More Cytotoxic Than Their β-Alkoxy Alcohol Counterparts

The biological activity of twelve compounds was tested against human gastric adenocarcinoma (AGS) and human lung adenocarcinoma (A549) cell lines. All molecules were initially evaluated at a single concentration (200 μM) to allow the identification of the most active ones. A549 cells are known to be resistant to chemotherapy, contrariwise to AGS, which are more responsive [15,16,17].

**Figure 1:**
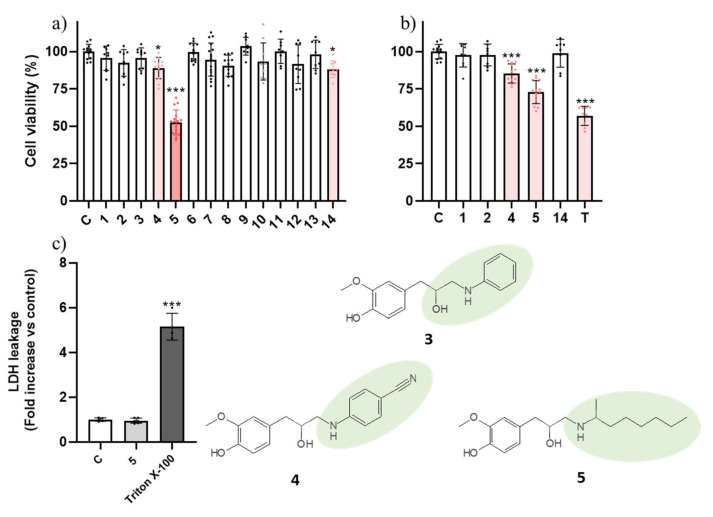
Viability of AGS cells exposed to the molecules at 200 μM (**a**) and 100 μM (**b**). The effect of topotecan at 5 μM is also presented in (**b**) for benchmark purposes. (**c**) Influence of molecule **5** (100 μM) on membrane integrity of AGS cells after 24 h. Triton X-100 at 1% was used as a positive control for maximum extracellular LDH activity. Each dot represents a single determination. C: control; T: topotecan. * *p* < 0.05; *** *p* < 0.001.

Earlier reports in the literature refer the toxicity of eugenol to AGS cells with 12 h of incubation [12]. In our conditions, no impact of eugenol (**1**) or its epoxide (**2**) upon cell viability was found (Figure 1a); however, this is likely related to the concentration used, 200 μM, while earlier reports used a much higher concentration (700 μM). By contrast, β-amino alcohols **4**, **5**, and **14** elicited a statistically significant reduction in cell viability (Figure 1a). When compared with compound **3**, which was inactive, the only difference in compound **4** is the presence of a benzonitrile substituent versus the benzyl substituent found in compound **3**, thus pointing to the importance of the cyanide group in the biological effect.

Substitution of the benzonitrile group by an aliphatic chain (**5**) markedly increased the toxicity of the molecule. These molecules were further tested at a lower concentration (100 µM) and they were still active (Figure 1b), with the exception of compound **14**, for which no impact on cell viability was found. Being the most potent molecule, compound **5** advanced into subsequent assessment.

**Figure 2:**
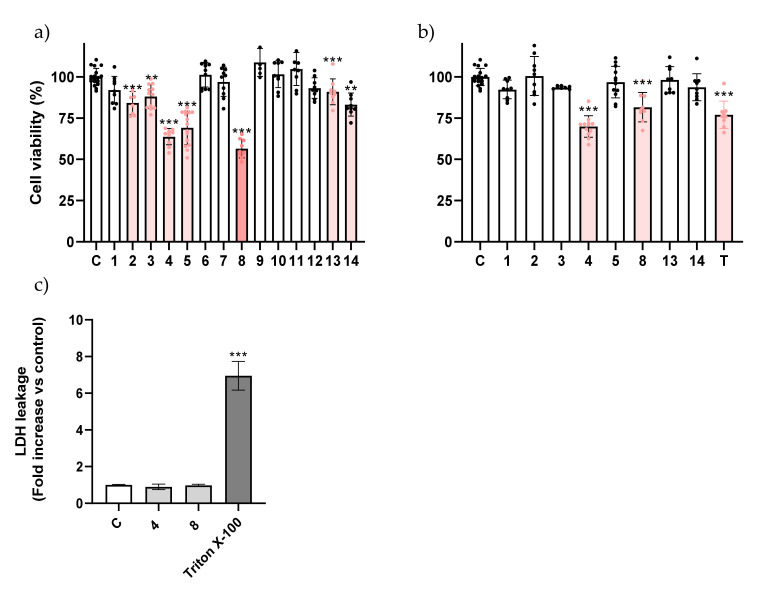
Viability of A549 cells exposed to the molecules at 200 μM (**a**) and 100 μM (**b**). The effect of topotecan at 5 μM is also presented in (**b**). (**c**) Influence of molecules **4** and **8** at 100 μM on membrane integrity of A549 cell line after 24 h. Triton X-100 at 1% was used as a positive control for maximum extracellular LDH activity. Each dot represents a single determination. C: control; T: topotecan. ** *p* < 0.01 *** *p* < 0.001.

In A549 cells, β-amino alcohols **3**, **4**, **5**, **8**, and **14** had more impact on reducing the cell viability than the β-alkoxy alcohols, with only one derivative of the latter group (**13**) presenting a significant impact on cell viability (Figure 2a). When the concentration of the compounds was reduced to 100 μM, compounds **4** and **8** continued to exhibit toxicity, a trait not found for the parent molecule at these concentrations (Figure 2b). Compound **8** is structurally very similar to the inactive molecule **3**, the difference being the methylation of the amine, which is thus highlighted as an important trait. For this reason, compounds **4**, **5**, and **8** were picked up to be further studied. Among all derivatives studied, the results clearly show that β-amino alcohols were the most cytotoxic subset of molecules.

After selecting the most active molecules of the series, we were interested in evaluating whether the impact on cell viability involved the loss of membrane integrity. Considering molecules that elicit necrosis are generally avoided in clinical practice, to find such a trait would reduce interest in the molecules. To this end, we assessed the impact of compound **5** on extracellular levels of lactate dehydrogenase (LDH) in AGS cells, a marker of plasma membrane integrity, and no impact was found, which suggests that compound **5** may cause a process of organized cell death (Figure 1c) [3]. As had been found in AGS cells, compounds **4** and **8** also did not negatively impact membrane integrity in A549 cells (Figure 2c). In light of this necrosis-independent capacity to reduce cancer cell viability, we hypothesized that these molecules could be triggering a process of programmed cell death such as apoptosis, a process during which the plasma membrane remains intact.

**Figure 3:**
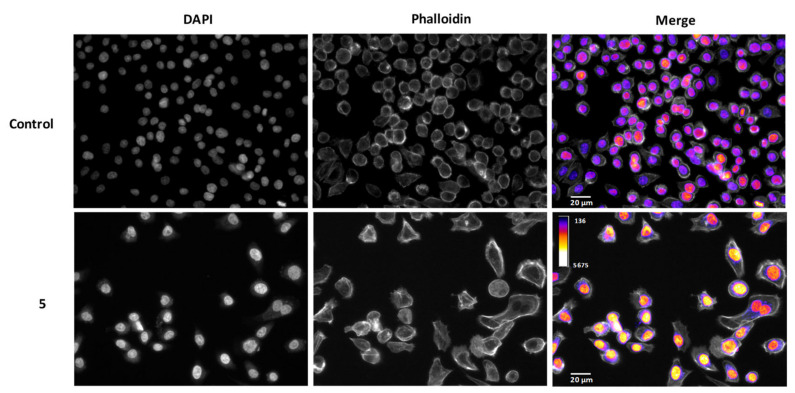
Morphological assessment of AGS cells incubated with compound **5** (100 µM, 24 h). DNA was studied using DAPI and overall cell morphology with phalloidin.

We took the most promising compound, **5**, and evaluated its impact upon cell morphology. As shown in Figure 3, incubation of AGS cells with compound **5** resulted in a clear reduction in cell density, in agreement with the results on cell viability. By using an intensity sensitive lookup table (LUT), it is clear that cells treated with compound **5** display significantly higher signals for 4’,6-diamidino-2-phenylindole (DAPI) when compared to the control, which points to chromatin condensation. In some fields, it was also possible to find chromatin fragmentation (Figure S1). These morphological characteristics indicated that programmed cell death, namely apoptosis, could be involved in cell-viability reduction.

### 2.2. The Toxicity of Eugenol Derivatives Is Selective for Cancer Cells

Considering the ability of the molecules presented to elicit cell death in cancer cells, we were interested in assessing whether this effect was selective. To this end, we evaluated their effect on noncancer cells, namely human keratinocytes (HaCaT). When tested at 100 μM for 24 h, the same conditions at which toxicity was found for cancer cells, neither of the molecules resulted in a loss of viability in noncancer cells, thus pointing to their selectivity (Figure S2).

### 2.3. Eugenol β-Amino Alcohols Activate Several Caspase Isoforms

As mentioned before, caspases are essential for the onset and development of apoptosis, with caspase-3 being the most relevant executor caspase isoform and caspases-8 and -9 typically classified as initiator caspases. In light of the impact on cell viability exhibited by the molecules presented so far, and considering that this effect was found to be non-necrotic, we hypothesized that a process of organized cell death could be taking place. To confirm this, we evaluated the effect of these compounds on the activity of several caspase isoforms. Topotecan, a topoisomerase I inhibitor that is well-characterized as an apoptosis activator [18], was used as positive control.

**Figure 4:**
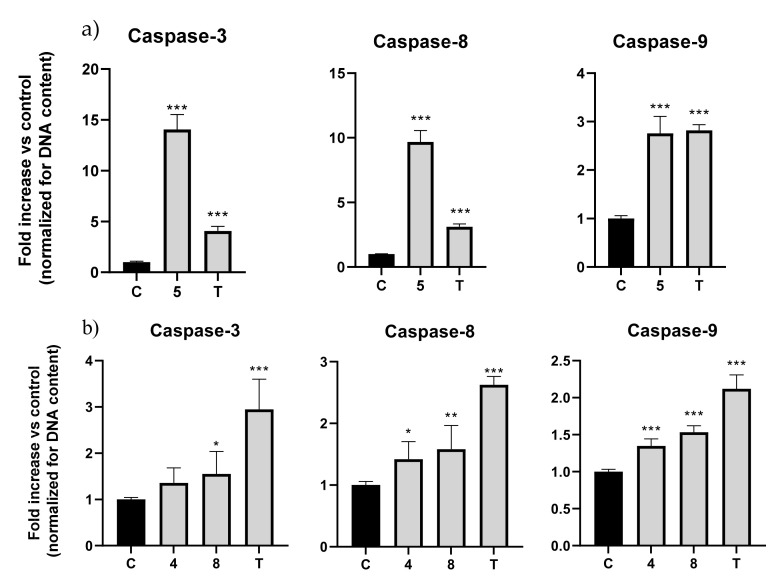
Fold increase (normalized for DNA content) in caspase-3, -8, and -9 activation in (**a**) AGS with molecule **5** and (**b**) A549 with molecules **4** and **8**. Topotecan at 2.5 μM was used as positive control. The values were normalized for DNA content; C: control; T: topotecan. * *p* < 0.05; ** *p* < 0.01; *** *p* < 0.001.

This experiment revealed that for the AGS cell line, molecule **5** elicited an increase in caspase-3, -8, and -9 activity. Caspase-3 increased 14 fold, caspase-8 nearly 10 fold, and lastly, caspase-9 increased 2.8 fold (Figure 4a). The results showed that molecule **5** in AGS cells could increase both initiator caspases-8 and -9, meaning that both the intrinsic and extrinsic pathways were activated, converging with the activation of executor caspase-3, as shown before [19].

Concerning the results for A549 cells, molecule **4** showed an increase in activity in caspase-3 of 1.35 fold, in caspase-8 of 1.41 fold, and in caspase-9 of 1.35 fold. Molecule **8** increased caspase-3 by 1.55 fold, caspase-8 by 1.58 fold, and lastly, caspase-9 increased by 1.54 fold (Figure 4b).

In general, all three molecules could induce the activation of caspases, with compound **5** activating with the greatest magnitude.

### 2.4. β-Amino Alcohol 5 Enhances the Toxicity of Topotecan

The parent molecule eugenol was previously reported as having the capacity to synergize with clinically used anticancer drugs, thus resulting in increased cytotoxic effects [10,20]. For that reason, we decided to study whether this feature was retained in its β-amino alcohol derivatives, for which we used the combination of compound **5** and topotecan, known to activate apoptosis, as mentioned before.

**Figure 5:**
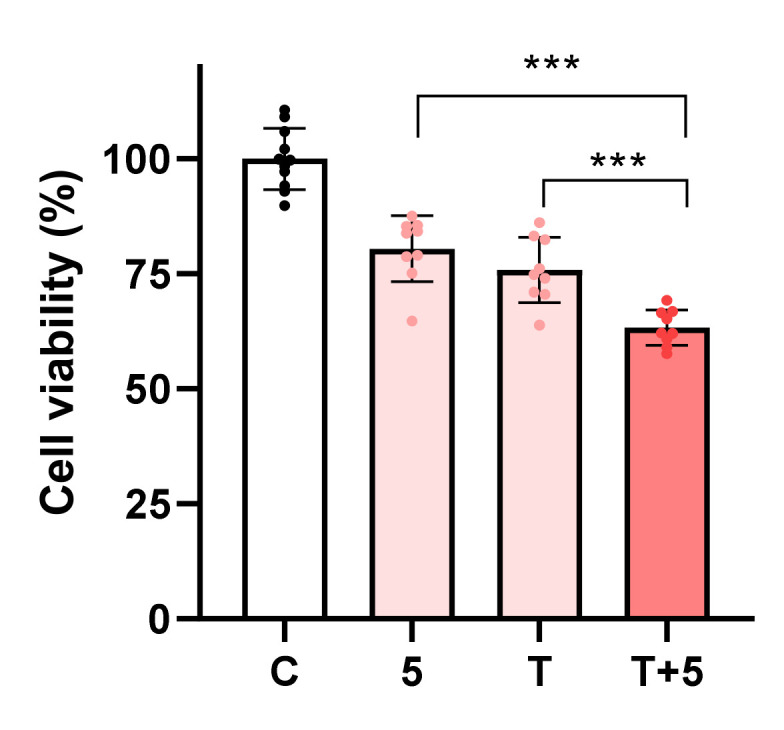
Viability of AGS cells exposed to molecule **5** at 100 μM combined with topotecan at 2.5 μM. Each dot represents a single determination. C: control; T: topotecan. *** *p* < 0.001.

Molecule **5** (100 μM) in combination with topotecan (2.5 μM) in AGS cells had a noticeable effect upon cell viability when compared with either molecule alone (Figure 5); therefore, this combination could be used to enhance the effect of the clinically used anticancer drug topotecan. In the case of molecules **4** and **8**, their combination with topotecan in A549 cells did not enhance cytotoxic activity (data not shown).

### 2.5. Derivatives ***5*** and ***8***, but Not ***4***, Have a Time-Dependent Effect

To understand whether the impacts of the molecules under study were time-dependent, compounds **4**, **5**, and **8** were additionally tested for their cytotoxic effects at 48 h and 72 h of incubation.

**Figure 6:**
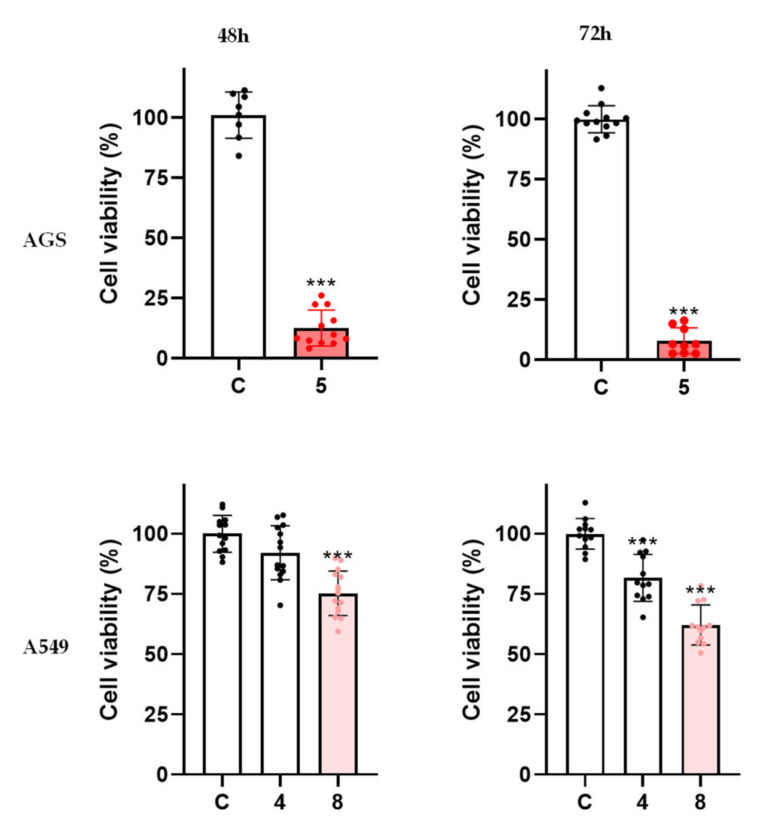
Viability of AGS cells exposed to molecule **5** (100 μM) at 48 h/72 h and viability of A549 cells exposed to molecules **4** and **8** (100 μM) at 48 h/72 h. The results correspond to the mean value for at least three independent experiments performed in triplicate. Each dot represents a single determination. C: control; *** *p* < 0.001.

In the case of compound **5**, the results show that at 48 h the inhibition of cell viability increased to 88% and at 72 h to 92% (Figure 6), a result markedly different than what had been found at 24 h (Figure 1b). As such, the effect of compound **5** on AGS cells is time-dependent.

Molecules **4** and **8** were also tested at 100 μM for a time-dependent effect in the A549 cell line. The results show that the effect of compound **4** did not change even with longer incubation periods, while a small decrease was found for compound **8**, namely a statistically significant increase in viability reduction from 24% to 37% (Figure 6). We observed that with longer exposure to compounds **5** and **8**, cytotoxic effects improved, so the effects of both molecules were shown to be time-dependent.

### 2.6. Structure-Activity Relationship

Considering the results, it was possible to conclude that the objective of obtaining molecules with more potent anticancer activity than the parent molecule, eugenol, was achieved. The first conclusion regarding the impact of the chemical structure upon cytotoxic activity is that all active molecules were β-amino alcohol derivatives, and thus this subclass is more biologically active than the β-alkoxy analogues.

The free hydroxyl group present in the benzene ring appears to be important to enhancing cytotoxic activity, as shown by a recent study which demonstrated that in a group of nitro-eugenol derivatives, the molecules containing a free hydroxyl group in their structure presented better cytotoxic effects [14]. Also, another study of several benzoate-eugenol derivatives against colon cancer cells (HT29) demonstrated that the two derivatives with free hydroxyl groups present in their carbon chains were some of the best compounds for providing cytotoxic effects. Additionally, the hydroxyl group was shown to be essential for developing apoptotic properties, namely in the case of the interaction with the Bcl-2 protein [21]. In the specific cases of the group of molecules evaluated herein, the free hydroxyl group is clearly not a pivotal factor for cytotoxicity as all molecules share this trait despite their distinct biological profiles.

When we compared β-amino-alcohols **3**, **4**, **8**, and **14**, which exhibited cytotoxic effects on A549 cells, it was possible to see that all molecules have an aromatic ring, namely benzene, attached to the three-carbon side chain, a trait not found in inactive molecules. While the inactive molecule **7** also shares this trait, its lack of activity may be associated with the methoxylation of the benzene ring, which is absent in all active molecules. Former studies have already proven that the attachment of an aromatic ring to the three-carbon side chain results in cytotoxic and chemopreventive molecules [7,22]. The same behavior was not verified in the AGS cells, which suggests a different manner of interaction between the molecules and the two cell lines.

When we compare compounds **3** and **4**, the only difference between them is the cyanide group in the benzyl substituent. This group must be playing a role, since molecule **4** was the only molecule with the benzyl substituent that had an impact on the AGS cells at a concentration of 100 μM. Additionally, in the A549 cells, the effects of molecule **4** remained at 100 μM but the same did not happen with molecule **3**. Analogues of paclitaxel, an approved chemotherapeutic drug with a cyanide group at the meta position on the C-2 benzoyl group proved to be more active than the parent molecule, although the same effect was not verified in the para position [23].

**Figure 7:**
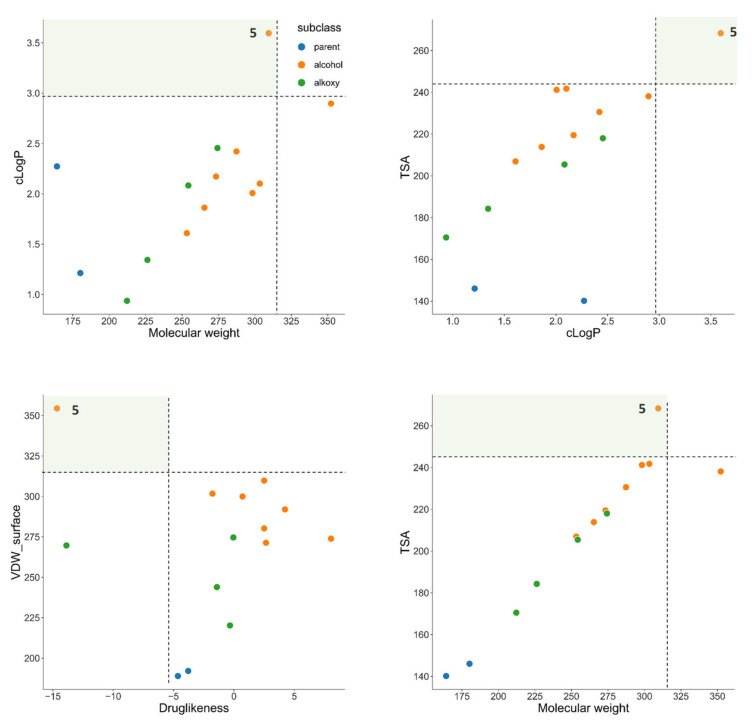
Chemical space occupied by the parent molecules **1** and **2** (blue dots) and β-amino (**3**–**9**, **14**—orange dots)/β-alkoxy (**10**–**13**—green dots) alcohol derivatives. cLogP, calculated partition coefficient; TSA, total surface area; VDW, van der Waals surface.

We also tried to understand whether the physicochemical properties of the molecules could be related to their activity. To this end, we calculated several properties of all molecules synthesized and tested (Table 1). Taking as example molecule **5**, which was the most potent molecule found in this evaluation, it was clear that when creating a 2D-chemical space consisting of distinct molecular descriptors (molecular weight, cLogP, TSA, VDW volume, and drug-likeness, among others), there are several combinations of descriptors in which compound **5** is clearly separated from the other molecules (Figure 7). This is a very important information as it shows that a chemometric analysis in tandem with structure−activity relationships and biological assessment can help us map and define the chemical spaces that are most likely to yield active molecules in the future. As such, the properties found here for the most potent molecule can guide future development of novel drugs by defining the ideal set of physicochemical properties.

## 3. Materials and Methods

### 3.1. Reagents

Dulbecco’s Modified Eagle Medium (DMEM), fetal bovine serum (FBS), 0.25% Trypsin-EDTA, Hanks’s balanced salt solution (HBSS), and penicillin/streptomycin solution were purchased from GIBCO (Invitrogen, NY, USA). Trypan blue, Triton X-100, dimethyl sulfoxide (DMSO), and topotecan hydrochloride hydrate were purchased from Sigma-Aldrich (Madrid, Spain). The 4’,6-diamidino-2-phenylindole (DAPI) was purchased from Sigma-Aldrich (St. Louis, MO, USA). Phalloidin was purchased from VWR. CytoTox 96^®^ Non-Radioactive Cytotoxicity Assay was purchased from Promega Corporation (WI, USA). PrestoBlue™ Cell Viability reagent and Qubit™ 1X dsDNA HS Assay Kit for DNA quantification was purchased from Thermo Fisher Scientific (Waltham, MA, USA). Caspase 3, Caspase 8, and Caspase 9 Multiplex Activity Assay Kit (Fluorometric) was purchased from Abcam (Cambridge, UK).

### 3.2. Synthesis of Eugenol Derivatives

**Figure 8:**
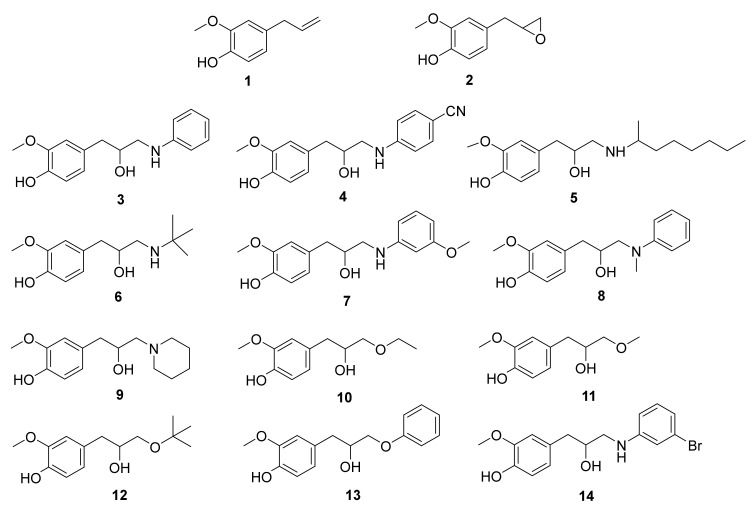
Chemical structures of eugenol **1**, epoxide **2**, β-amino alcohols (**3**–**9**, **14**) and β-alkoxy alcohols (**10**–**13**), obtained through different reactions between eugenol epoxide **2** and several amines or alcohols, respectively.

Firstly, eugenol **1** was obtained from *Syzygium aromaticum* (L.) Merr. & L.M. Perry through hydrodistillation, and then its epoxide **2** was prepared by reaction of eugenol **1** with *m*-chloroperoxybenzoic acid in dichloromethane using a known procedure [24].The reaction of eugenol epoxide **2** at 50 °C in ethanol and water with aniline, 4-cyanoaniline, octan-2-methylpropan-2-amine, 2-methylpropan-2-amine, 3-methoxyaniline, *N*-methylaniline, piperidine, and 3-bromoaniline, gave the eugenol β-amino alcohols **3**, **4**, **5**, **6**, **7**, **8**, **9**, and **14**, respectively, as we previously reported [25]. In addition, reaction of eugenol epoxide **2**, under nitrogen atmosphere, at 0 °C, using boron trifluoride diethyl etherate with ethanol, methanol, *tert*-butanol, and phenol afforded eugenol alkoxy alcohols **10**, **11**, **12**, and **13**, respectively (Figure 8) [26]. All molecules were purified by column chromatography and ^1^H NMR spectra are in agreement with those obtained previously for the same compounds and confirm the desired chemical structures [26].

### 3.3. Cell Culture

Human gastric adenocarcinoma (AGS; Sigma-Aldrich), human lung adenocarcinoma (A549; ECACC, Salisbury, UK) and human skin keratinocyte (HaCaT; ATCC, Rockville, MD, USA) cells were maintained with DMEM + GlutaMAX™ supplemented with 10% FBS and 1% penicillin/streptomycin at 37 °C in a humidified atmosphere of 5% CO_2_.

### 3.4. Cell Viability Assessment

For the assessment of cell viability, a resazurin-based method was used. Cells were seeded in 96-well plates, A549 at a density of 10,000 cells/well at 24 h, 5000 cells/well at 48 h, and 2500 cells/well at 72 h, while AGS were seeded at a density of 15,000 cells/well at 24 h, 10,000 cells/well at 48 h, and 5000 cells/well at 72 h, and HaCaT cells were seeded at 15,000 cells/well at 24 h. Cells were allowed to attach for 24 h and then exposed to the molecules under study for 24 h, 48 h, and 72 h. After this incubation period, PrestoBlue reagent was added (1:10) and incubated for 30 min. Finally, fluorescence was read at 560/590 nm (excitation/emission wavelength) in the SYNERGY H1 microplate reader. The results of cell viability correspond to the mean ± standard deviation (SD) of at least three independent experiments performed in triplicate. The percentage of viability of each well was measured in relation to the untreated cells (control group). Blank readings were subtracted from the values of treated and control groups.

### 3.5. LDH Assay

AGS and A549 cells were cultured in the presence of the molecules under study at the same density described above for the viability assay. After that, 30 μL of culture media were removed from each well and the LDH release was measured using CytoTox 96^®^ non-radioactive cytotoxicity assay kit from Promega according to the manufacturer’s instructions. The absorbance was read at 490 nm in the Thermo Scientific™ Multiskan™ GO microplate reader. Triton X-100 at 1% was used as positive control. The results correspond to the fold-increase in absorbance in treated versus untreated cells for three independent experiments performed in duplicate.

### 3.6. Morphology Assement

Cells were incubated in the presence of each compound for 24 h, as described before. After incubation, the medium was removed and cells were washed with HBSS and fixed with 10% formalin solution for 20 min at room temperature. Ending this period, wells were again washed with HBSS and incubated with DAPI (0.25 µg/mL) and phalloidin (0.5 µg/mL) for 20 min. Finally, the wells were washed three times with HBSS and left with 100 μL of this solution remaining in each well. Images were analyzed with Fiji. Two independent experiments were each performed in duplicate.

### 3.7. Caspase Activity Assay

AGS and A549 cells were plated at the same density described before and exposed to the molecules under study for 24 h. Caspase-3, -8, and -9 activity was assessed by using Abcam’s Caspase Multiplex Activity Assay Kit (Fluorometric). Cells were incubated in the presence of each compound for 24 h, as described before. After the incubation time, 50 μL of supernatant were removed from each well, followed by the addition of 50 μL of caspase substrate diluted at 1:200 in assay buffer. The plate was incubated for 45 min in a humidified incubator at 37 °C with 5% CO_2_, protected from light. After that, fluorescence was read in Cytation 3 at three specific wavelengths: caspase-3 (535–620 nm), caspase-8 (490–525 nm), and caspase-9 (370–450 nm). Topotecan was used as positive control, and all results were normalized for DNA content to account for potential differences in cell density arising from cell death. The results correspond to the fold-increase in fluorescence in treated versus untreated cells in each well in at least three independent experiments performed in duplicate.

### 3.8. DNA Quantification

Cells were cultured and exposed to the molecules under study, as referred before. Past the incubation period, the culture medium was replaced by 50 μL of ultra-pure water and then the plate was incubated for 30 min in a humidified incubator at 37 °C with 5% CO_2_ and subsequently frozen at −80 °C. DNA quantification was performed by using Qubit^TM^ 1X dsDNA HS assay kit according to the manufacturer’s instructions. Three independent experiments were performed in duplicate. The quantification was performed in order to normalize the results from the caspase activity assay.

### 3.9. Chemometric Analysis

The properties and descriptors of the molecules were calculated using DataWarrior (https://openmolecules.org/datawarrior/, accessed on 20 January 2022). The data was exported to a csv file and analysed using Phyton 3.9, Numpy, and Pandas. Graphics for Figure 7 were generated using Seaborn 0.11.2.

### 3.10. Statistical Analysis

Normality tests were performed for the data, which was shown to follow a normal distribution. As such, results were statistically analyzed using the one-way ANOVA to determine whether the treated groups were significantly different from the untreated group (control). After that, Dunnett’s test was performed to know which groups were significantly different, and a *p* < 0.05 was considered statistically significant (* *p* < 0.05; ** *p* < 0.01; *** *p* < 0.001). Data are expressed as mean ± SD. All statistical analyses were performed through the GraphPad Prism 8.4.2 software.

## 4. Conclusions

Several β-amino-alcohol and β-alkoxy-alcohol eugenol derivatives were studied for their cytotoxic properties against AGS and A549 cells. The results showed that some derivatives possess enhanced cytotoxic properties in relation to eugenol. Additionally, it was possible to understand that β-amino-alcohol derivatives were more cytotoxic to A549 and AGS cells when compared with β-alkoxy-alcohol derivatives and the parent molecule.

Also, we identified that β-amino alcohols (**4** and **8**) with a benzene ring attached to the three-carbon chain have more impact on A549 cells, showing that AGS and A549 cells interact in different ways with the molecules. With regard to AGS cells, compound **5** with an aliphatic chain instead of a benzene ring attached to the three-carbon chain had impact on cell viability. Additionally, compound **5** was the most potent molecule and stands out from the other molecules in several descriptor combinations which give us clues to determine new molecules with specific physicochemical characteristics. We further clarified that cytotoxic proprieties of compounds **4**, **5**, and **8** were related to the activation of apoptosis, once they trigger caspases-3, -8, and -9, and morphologic assessment of compound **5** demonstrated chromatin condensation and fragmentation, which are morphological characteristics of the apoptotic process. Moreover, compounds **4**, **5**, and **8** were shown to be selective for cancerous cells at 100 μM.

For subsequent studies, understanding the interactions between the molecules and their targets via in silico studies, such as inverse virtual screening, could result in new and more potent anticancer molecules.

## Figures and Tables

**Table 1:** Calculated properties of the molecules under study. Interm: intermediate; PSA: polar surface area; VDW: van der Walls.

Molecule	1	2	3	4	5	6	7	8	9	14	10	11	12	13
Group	Parent/Interm		β-Amino Alcohols								β-Alkoxy Alcohols			
**Molecular weight**	164.20	180.20	273.33	298.34	309.45	253.34	303.36	287.36	265.35	352.23	226.27	212.24	254.33	274.32
**cLogP**	2.27	1.21	2.17	2.01	3.60	1.61	2.10	2.42	1.86	2.90	1.34	0.94	2.08	2.45
**cLogS**	−2.05	−1.686	−2.704	−3.477	−3.117	−1.984	−2.722	−2.68	−1.791	−3.538	−1.633	−1.333	−2.12	−2.662
**H-Acceptors**	2	3	4	5	4	4	5	4	4	4	4	4	4	4
**H-Donors**	1	1	3	3	3	3	3	2	2	3	2	2	2	2
**Total Surface Area**	140.17	146.04	219.46	241.17	265.52	206.89	241.72	230.56	213.84	238.09	184.26	170.5	205.43	218
**Relative PSA**	0.16	0.28	0.22	0.25	0.18	0.23	0.24	0.17	0.19	0.20	0.25	0.27	0.22	0.21
**Polar Surface Area**	29.46	41.99	61.72	85.51	61.72	61.72	70.95	52.93	52.93	61.72	58.92	58.92	58.92	58.92
**Druglikeness**	−4.64	−3.79	2.50	−1.78	−7.76	8.04	2.50	4.23	2.66	0.71	−1.41	−0.33	−13.87	−0.05
**Non-H Atoms**	12	13	20	22	22	18	22	21	19	21	16	15	18	20
**Non-C/H Atoms**	2	3	4	5	4	4	5	4	4	5	4	4	4	4
**Electronegative Atoms**	2	3	4	5	4	4	5	4	4	5	4	4	4	4
**Stereo Centers**	0	1	1	1	1	1	1	1	1	1	1	1	1	1
**Rotatable Bonds**	3	3	6	6	11	6	7	6	5	6	6	5	6	6
**Aromatic Atoms**	6	6	12	12	6	6	12	12	6	12	6	6	6	12
**sp3-Atoms**	4	7	7	7	16	12	9	8	13	7	10	9	12	8
**Carbo-Rings**	1	1	2	2	1	1	2	2	1	2	1	1	1	2
**Hetero-Rings**	0	1	0	0	0	0	0	0	1	0	0	0	0	0
**Non-Aromatic Rings**	0	1	0	0	0	0	0	0	1	0	0	0	0	0
**Aromatic Rings**	1	1	2	2	1	1	2	2	1	2	1	1	1	2
**Amines**	0	0	1	1	1	1	1	1	1	1	0	0	0	0
**Alkyl-Amines**	0	0	0	0	1	1	0	0	1	0	0	0	0	0
**Aromatic Amines**	0	0	1	1	0	0	1	1	0	1	0	0	0	0
**Basic Nitrogens**	0	0	0	0	1	1	0	0	1	0	0	0	0	0
**VDW-Surface**	188.95	192.07	280.22	301.71	343.78	273.89	309.8	291.99	271.35	299.96	243.92	220.22	269.69	274.62
**VDW-Volume**	174.15	179.9	280.28	300.96	347.12	276.4	307.53	301.51	281.39	299.68	233.78	215.05	273.74	273.89

## Data Availability

Not applicable.

## Supplementary Materials

The following supporting information can be downloaded at: https://www.mdpi.com/article/10.3390/ijms23073759/s1.
