# Supplementary material for: Eugenol β-Amino/β-Alkoxy Alcohols with Selective Anticancer Activity
Source: Int J Mol Sci. 2022 Mar 29;23(7):3759. doi: 10.3390/ijms23073759 (PMC8999083; doi:10.3390/ijms23073759)
Supplement: Supplementary file 1 [file ijms-23-03759-s001.zip › ijms-1633318-supplementary.pdf]

Supplementary Material

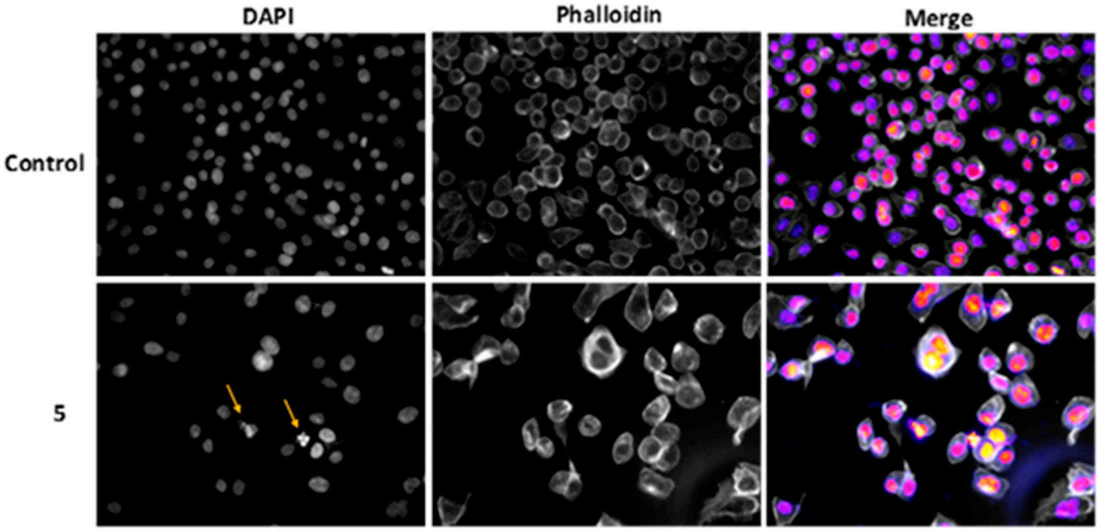

Figure S1 - Morphological assessment of AGS cells incubated with **5** (100 µM, 24h). DNA was studied using DAPI and overall cell morphology with phalloidin. Yellow arrows: chromatin fragmentation.

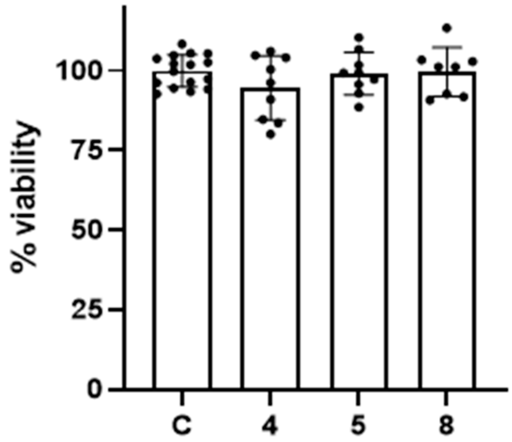

Figure S2 - Viability of HaCaT cells exposed to molecules **4**, **5** and **8** at 100 µM. The results correspond to the mean of each well of three independent experiments performed in triplicate. C: control.
